# Peripheral immunity is associated with cognitive impairment after acute minor ischemic stroke and transient ischemic attack

**DOI:** 10.1038/s41598-024-67172-w

**Published:** 2024-07-13

**Authors:** PanPan Zhao, GuiMei Zhang, YongChun Wang, ChunXiao Wei, ZiCheng Wang, WeiJie Zhai, YanXin Shen, Lin Shi, Li Sun

**Affiliations:** Department of Neurology and Neuroscience Center, The First Hospital of Jilin University, Jilin University, Xinmin Street 71#, Changchun, 130021 China

**Keywords:** Lymphocytes, Neutrophils, Neutrophil-to-lympho cyte ratio, Peripheral immunity, Post-stroke cognitive impairment, Predictive model, Immunology, Neuroscience, Biomarkers, Neurology

## Abstract

Immunoinflammation is associated with the development of post-stroke cognitive impairment (PSCI), however, peripheral immunity has not been fully explored. We aimed to investigate the association between PSCI and peripheral immune indicators, including neutrophil, lymphocyte, and mononuclear percentages and counts; the systemic immune inflammation index; platelet-to-lymphocyte ratio; neutrophil-to-lymphocyte ratio (NLR); and lymphocyte-to-monocyte ratio. A total of 224 patients with acute minor ischemic stroke or transient ischemic attack with 6–12 months of follow-up were included. PSCI was defined as a Montreal Cognitive Assessment score < 22 during the follow-up period. We performed logistic regression, subgroup analyses based on age and sex, and further established predictive models. We found that increased innate immunity indicators (neutrophils, neutrophil percentage) increased the risk of PSCI, whereas increased adaptive immunity indicator (lymphocytes) were protective against PSCI, especially in patients aged 50–65 years. Neutrophil percentage and NLR improved the predictive efficacy of the models that included demographic, clinical, and imaging information, with the area under the curve increased from 0.765 to 0.804 and 0.803 (*P* = 0.042 and 0.049, respectively). We conducted a comprehensive analysis of peripheral immunity in PSCI, providing a novel perspective on the early detection, etiology, and treatment of PSCI.

## Introduction

In 2020, 89.13 million cases of stroke were reported worldwide, with ischemic stroke accounting for 68.16 million cases. ^1^ Post-stroke cognitive impairment (PSCI) refers to any degree of cognitive deficit that occurs following an overt stroke, ^2^ and the PSCI incidence rates have been reported to be as high as 70% depending on stroke type and time of evaluation.^3^ PSCI increases disability and mortality after stroke^1^ and is also associated with stroke recurrence.^4^ The impairment of cognitive domain varies in different stroke types. The impairment of executive function, attention, memory and language function after ischemic events is common. The impairment of cognitive domain after hemorrhagic events is similar to that of ischemic events, depending on the regions of injury, and after subarachnoid hemorrhage impairments in executive function and verbal memory account for the highest proportion.^2^ These impairments result in significant direct and indirect social and economic burdens worldwide.^1^ Unlike severe stroke, minor stroke and transient ischemic attack (TIA) often do not receive sufficient attention from patients due to the generally short duration of their symptoms. Many studies have shown that TIA and minor stroke may cause delayed brain atrophy and cognitive disorders^3,5–7^. In one Oxford Vascular study, the incidence rates of dementia were 8.2% and 5% 1 year after a minor stroke (National Institutes of Health Stroke Scale [NIHSS] score < 3) and after TIA, respectively, and the risk of dementia advanced by approximately 4 years in patients with minor stroke, and 2 years in TIA, when compared to age- and sex-matched populations in the UK^7^. Owing to the complex etiology and pathology of PSCI, there are no available Food and Drug Administration-approved drugs.

It is well known that vascular risk factors and degeneration are all involved in the pathogenesis of PSCI^2,3,8^. However, stroke^9,10^ and cognitive impairment^11^ occurred in people without vascular disease risk factors after infection with the worldwide coronavirus disease 2019 (COVID-19) pandemic, suggesting that systemic immunoinflammation plays a key role in the development of stroke and cognitive disorders^10,12^. Moreover, systemic inflammation^13^, including altered levels of peripheral blood complement system, interleukins, and chemokines ^14^, and neuroinflammation^15^ triggered by acute stroke have all been shown to be involved in the pathological process of PSCI^10,14,16^. However, the immune system is a double-edged sword in the pathophysiology of stroke^17^. Once acute cerebral ischemia occurs, damage-associated molecular patterns are released, triggering the brain’s intrinsic immune cells (microglia) and recruiting infiltration of peripheral innate immune cells, including neutrophils ^10,18^ and mononuclear macrophages^12^, leading to increased ischemic injury, but it may also have a protective effect^17^. Meanwhile, systemic immune activation followed by severe immune suppression can promote infection^17^. In the chronic phase, antigen presentation initiates an adaptive immune response against the brain, contributing to post-stroke complications such as depression and cognitive impairment^17,19,20^.

The most commonly used methods to evaluate peripheral immunity include the measurement of blood neutrophils, lymphocyte, and mononuclear percentages and counts, and their derived ratios, such as the systemic immune inflammation index (SII), platelet-to-lymphocyte ratio (PLR), neutrophil-to-lymphocyte ratio (NLR), and lymphocyte-to-monocyte ratio (LMR)^21^. and neutrophils, monocytes, SII, PLR, NLR represent peripheral innate immunity, while lymphocytes and LMR represent peripheral adaptive immunity^21^. To date, no systematic research is available on peripheral immunity and PSCI, and the contributions of peripheral innate and adaptive immune events to the pathogenesis of PSCI remain unclear^19,20,22^. However, early interventions targeting PSCI and related risk factors is essential to prevent further worsening of cognitive decline^3,23,24^. Therefore, this study aimed to further explore the correlation between peripheral immunity and cognitive function 6–12 months after acute stroke and TIA events, to explore the possible mechanisms underlying PSCI, and to establish a prediction model to provide a basis for further follow-up, diagnosis, and treatment of patients with possible PSCI.

## Methods

### Study population

**Figure 1:**
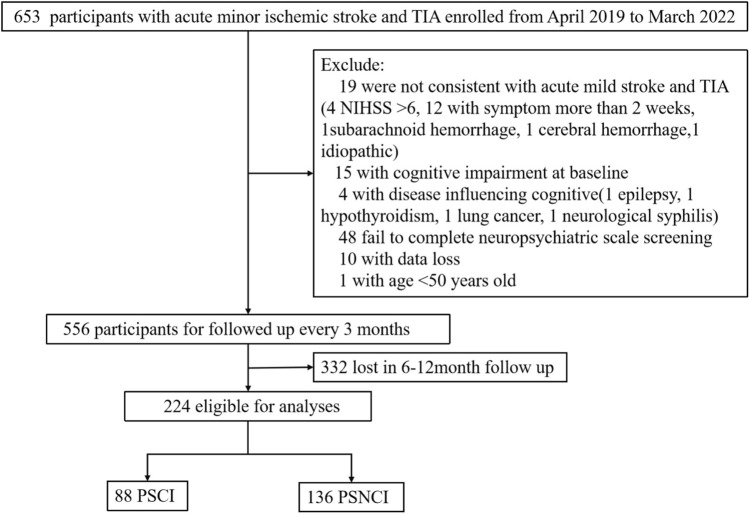
Flowchart of participant selection, NIHSS = National Institutes of Health Stroke Scale, PSCI = post-stroke cognitive impairment, PSCIN = post-stroke no cognitive impairment, TIA = transient ischemic attack.

In total, 653 patients with acute minor ischemic stroke and TIA admitted to the Department of Neurology of the First Hospital of Jilin University were prospectively enrolled in this study between April 2019 and March 2022. A previous article comprehensively described the inclusion and exclusion criteria^25^. The inclusion criteria were: (1) satisfying the WHO diagnostic criteria for TIA and acute ischemic stroke, (2) age 50–80 years, and (3) NIHSS score ≤ 6. The exclusion criteria were: (1) use of medications that affect cognition within 2 weeks or a history of cognitive disorders; (2) inability to cooperate with cognitive assessment; and (3) disease and treatment within 2 weeks that may affect peripheral immunity. Based on the inclusion and exclusion criteria, 556 patients were screened for follow-up at 3-month intervals, and data from 224 patients who were followed up for 6–12 months were further analyzed (Fig. 1).

## Ethics approval

The research was carried out following the World Medical Association Declaration of Helsinki and was approved by the First Hospital of Jilin University Ethics Committee (protocol code 19K023-003). The procedures followed conformed to national and institutional guidelines, and written informed consent was obtained from all participants. This study was registered with the Chinese Clinical Trial Registry (URL: https://www.chictr.org.cn/; unique identifier: ChiCTR1900022675).

## Study variables

We collected baseline demographic data (age, sex, and years of education), medical history (smoking, alcohol consumption, hypertension, diabetes, stroke, and coronary artery disease), clinical information (diastolic blood pressure, systolic blood pressure, height, and weight), and laboratory test findings (peripheral blood cell count, lipid level, glucose level, uric acid level, and homocysteine level). Years of education were divided into three levels: ≤ 6 years, 6–12 years, and > 12 years. Diabetes mellitus was defined as a history of diabetes or admission with fasting blood glucose level ≥ 6.1 and glycosylated hemoglobin level > 6 or random blood glucose level ≥ 7.8 and glycosylated hemoglobin level > 6 or glycosylated hemoglobin level alone ≥ 6.5.^26^ Hypertension was defined as a history of hypertension or admission with systolic blood pressure ≥ 140 mmHg and diastolic blood pressure ≥ 90 mmHg. Coronary artery disease and stroke were classified as yes or no based on previous history of the conditions. Smoking was classified as never smoking, former smoking, and current smoking. Former smoking refers to smoking cessation for > 1 year or opportunistic smoking. Alcohol consumption was categorized as never drinking, former drinking, and current drinking, and former drinking refers to abstinence for > 1 year or opportunistic drinking.

Stroke subtypes were classified by the Trial of Org 10,172 in Acute Stroke Treatment (TOAST).^27^ An NIHSS score^28^ of 0–6 was used to evaluate the severity of neurological impairment on admission. White matter hypertension, including Fazekas scores, deep white matter hyperintensity (DWMH), and periventricular hyperintensity (PVH) based on Flair axial brain magnetic resonance imaging (MRI)^29^ were assessed by an imaging physician within 72 h of stroke/TIA onset.

Using peripheral blood cells obtained within 24 h of symptom onset, we measured the following parameters: white blood cell counts including leukocyte, neutrophil, lymphocyte, and mononuclear percentage; neutrophil, lymphocyte, and mononuclear values; platelet count; and their associated ratios (NLR, LMR, PLR, and SII). The remaining hematological parameters were obtained after morning fasting on the day of enrollment. Cell counts were analyzed using a hematology analyzer (SYSMEX XN-9000, Kobe, Japan), and biochemical indices were determined using a chemistry analyzer (AU480, Beckman Coulter, Brea, CA, USA).

## Cognitive assessment and outcome measurement

Patients with a history of cognitive impairment were excluded upon enrollment. The enrolled patients completed the first baseline scale assessment, including the Montreal Cognitive Assessment (MoCA) and Mini-Mental State Examination (MMSE), when their condition was stable 7–10 days after admission. Subsequently, patients were assessed every 3 months using the above neuropsychiatric scales, and those who underwent follow-up at 6–12 months were analyzed according to the definition of PSCI. If the patients underwent > 1 follow-up within 12 months, the information from the last scale was taken, and face-to-face follow-up scale information was prioritized when video scale information was also available during this period. These scales were scored by a full-time assessor with more than 5 years of experience, and 1 point was added to the total MMSE/MoCA score for years of education < 12. Previous studies have shown good sensitivity and specificity of the MoCA for PSCI assessment.^30^ We used the MoCA as the outcome rating scale and defined the follow-up score of MoCA < 22 as PSCI and MoCA ≥ 22 as post-stroke no cognitive impairment (PSNCI).^30^

## Statistical analysis

The patients’ baseline characteristics, such as age and other continuous variables, are presented as medians and interquartile ranges, whereas categorical variables such as previous stroke are described using counts and percentages. First, the Mann–Whitney U test (for continuous variables) and chi-square test (for categorical variables) were conducted to analyze the bias between patients with and without follow-up. Subsequently, Mann–Whitney U test (for continuous variables), chi-square test (for categorical variables), and univariate logistic regression analyses were separately employed on patients with and without PSCI in the follow-up group. For statistically significant indicators of peripheral immunity in univariate logistic regression, we dichotomized neutrophils, lymphocyte percentage, neutrophil values, NLR, and SII based on optimal cut-off values (72.5%, 33.5%, 5.01 × 109/L, 3.875, and 767.3, respectively) obtained from receiver operating characteristic (ROC) curves (Supplementary Fig. S1). We further adjusted for age, sex, height, hypertension, previous stroke, systolic blood pressure, years of education, the NIHSS score, DWMH score, and Fazekas score using multivariate binary logistic regression analysis. Subgroup analyses were performed based on age and sex. Finally, the prediction model was constructed according to the baseline variables for which *P* < 0.05 in the univariate logistic regression analysis were considered clinically relevant using stepwise backward multivariate logistic regression (Supplementary Table S1). The ROC curve was used to quantify the model’s predictive value, and the Wilcoxon signed-rank test was used to compare the area under the ROC curves. The missing values, all for continuous variables and accounting for less than 7%, were imputed using the median of baseline data (Supplementary Table S2). All statistical analyses were performed using SPSS version 26 (IBM Corp), and a two-sided *P* value < 0.05 was considered statistical significance. Graphical representations were created using GraphPad Prism9 (GraphPad Software).

## Results

### Population characteristics

A total of 224 of the 556 participants were followed up between 6 and 12 months after discharge, of which 19 were diagnosed with TIA, and the rest 205 were diagnosed with minor stroke. Among the participants who were followed up, 88 (39.28%) were diagnosed with PSCI, of which 3 were diagnosed with TIA, and 83 (94.3) were followed up through a face-to-face visit. Among the follow-up patients, 75% were men, with a median age of 61 years and a median education duration of 12 years; 23.7% had a previous stroke, and the median NIHSS score was 2. Compared with patients in the PSNCI group, those in the PSCI group were more often women (35.2% vs. 18.4%, *P* = 0.004), had fewer years of education (median 9 vs. 12 years, *P* < 0.001), were shorter in height (median 168 vs. 170 cm, *P* = 0.014), had higher rates of hypertension (89.8% vs. 75%, *P* = 0.006) and previous stroke (30.7% vs. 19.1%, *P* = 0.047), had higher systolic blood pressure on admission (median 147.5 vs. 140.5 mmHg, *P* < 0.01), had more severe neurological deficits and white matter lesions (the *P* values for the NIHSS, DWMH, and Fazekas scores were 0.02, 0.008, and 0.039, respectively), and had higher SII scores (median 583.12 vs. 471.05, *P* = 0.028). Details of the follow-up patients can be found in Table 1.

**Table 1:** Baseline characteristics of the follow-up patients by cognitive status.

	Total	PSCI	PSNCI	Statistics		Univariate logistic regression	
	n = 224	n = 88	n = 136	Z/χ^2^	*P*	OR(95%CI)	*P*
Age(years)	61(56–68)	62(57.25–69)	60.5(55–67)	− 1.957	0.050	1.039(1.001–1.078)	0.045*
≥ 65(%)	83(37.1)	35(39.8)	48(35.3)	0.459	0.498	1.211(0.696–2.105)	0.498
Male(%)	168(75)	57(64.8)	111(81.6)	8.086	0.004**	2.415(1.304–4.471)	0.005**
Education(years)	12(9–15)	9(6–12)	12(9–15)	− 4.796	< 0.001***	0.817(0.751–0.89)	< 0.001***
Education level				21.147	< 0.001***		
< = 6y	45(20.1)	28(31.8)	17(12.5)				
6-12y	119(53.1)	49(55.7)	70(51.5)			0.425(0.21–0.86)	0.017*
> 12y	60(26.8)	11(12.5)	49(36)			0.136(0.056–0.332)	< 0.001***
Height(cm)	169.5(162–173)	168(160–172)	170(165–173.75)	− 2.45	0.014*	0.947(0.911–0.984)	0.005**
Weight(kg)	70(63–80)	69.5(60.5–75.75)	70(65–80)	− 1.758	0.079	0.977(0.953–1.002)	0.073
BMI(kg/m^2^)	24.87(23.02–27.09)	24.85(23.34–27.04)	24.87(22.72–27.31)	− 0.271	0.786	0.992(0.909–1.082)	0.856
Hypertension(%)	181(80.8)	79(89.8)	102(75)	7.517	0.006**	2.926(1.326–6.455)	0.008**
Diabetes(%)	91(40.6)	34(38.6)	57(41.9)	0.238	0.626	0.873(0.505–1.509)	0.626
Coronary heart disease (%)	40(17.9)	14(15.9)	26(19.1)	0.375	0.540	0.8(0.392–1.634)	0.541
Previous stroke (%)	53(23.7)	27(30.7)	26(19.1)	3.956	0.047*	1.873(1.005–3.491)	0.048*
Smoking (%)				0.078	0.962		
Never	115(51.3)	46(52.3)	69(50.7)				
Previous	19(8.5)	7(8)	12(8.8)			0.875(0.321–2.388)	0.794
Now	90(40.2)	35(39.8)	55(40.4)			0.955(0.543–1.679)	0.872
Alcohol Consumption(%)				3.633	0.163		
Never	125(55.8)	56(63.6)	69(50.7)				
Previous	39(17.4)	13(14.8)	26(19.1)			0.616(0.29–1.309)	0.208
Now	60(26.8)	19(21.6)	41(30.1)			0.571(0.299–1.092)	0.090
Systolic pressure (mmHg)	143(131–159)	147.5(136–160)	140.5(129.25–153)	− 2.579	< 0.01**	1.015(1.002–1.029)	0.026*
Diastolic pressure (mmHg)	85(76–94)	86.5(77.25–96)	83.5(76–91.75)	− 1.218	0.223	1.014(0.992–1.036)	0.214
Diagnose				4.805	0.028*		
TIA	19(8.5)	3(3.4)	16(11.8)				
Stroke	205(91.5)	85(96.6)	120(88.2)			3.778(1.067–13.372)	0.039*
NIHSS score	2(1–3)	2(1–3)	2(1–3)	–2.333	0.020*	1.241(1.032–1.492)	0.022*
TOAST				10.992	0.004^a^**		
LAA (%)	69(30.8)	28(31.8)	41(30.1)				
Cardioembolism (%)	1(0.4)	0(0)	1(0.7)				1.000
Small-vessel occlusion (%)	122(54.5)	39(44.3)	83(61)			0.688(0.373–1.27)	0.232
Others (%)	32(14.3)	21(23.9)	11(8.1)			2.795(1.167–6.696)	0.021*
Fazekas score	2(1–3)	2(2–4)	2(1–3)	− 2.066	0.039*	1.248(1.029–1.514)	0.024*
PVH score	1(1–2)	1(1–2)	1(1–1.75)	− 1.230	0.219	1.334(0.913–1.948)	0.136
DWMH score	1(0–1)	1(1–2)	1(0–1)	− 2.646	0.008**	1.613(1.137–2.288)	0.007**
Cyctatin C (mg/l)	0.95(0.86–1.08)	0.95(0.82–1.08)	0.95(0.87–1.08)	− 0.58	0.562	0.643(0.176–2.353)	0.505
FBG (mmol/L)	5.505(5.03–6.83)	5.37(4.97–6.90)	5.59(5.12–6.81)	− 0.632	0.527	0.898(0.672–1.2)	0.467
Total cholesterol(mmol/L)	4.5(3.79–5.12)	4.54(3.89–5.15)	4.46(3.67–5.05)	− 1.141	0.254	1.212(0.952–1.542)	0.119
Triglyceride (mmol/L)	1.49(1.09–1.91)	1.54(1.09–1.93)	1.48(1.1–1.9)	− 0.393	0.695	1.047(0.854–1.283)	0.66
HDL (mmol/L)	0.98(0.87–1.17)	1(0.89–1.188)	0.97(0.86–1.15)	− 1.161	0.246	1.474(0.485–4.479)	0.494
LDL (mmol/L)	2.785(2.35–3.42)	2.93(2.37–3.54)	2.69(2.33–3.37)	− 1.611	0.107	1.334(0.98–1.815)	0.067
Uric acid (umol/L)	314(270.5–380)	304.5(263.25–360)	319.5(278–388.75)	− 1.752	0.080	0.997(0.994–1.001)	0.111
Homocysteine (umol/L)	11.8(9.81–15.85)	11.8(9.95–16.1)	11.7(9.64–15.6)	− 0.456	0.648	1.006(0.985–1.027)	0.599
Glycosylated hemoglobin	6.1(5.6–6.98)	6.1(5.6–6.78)	6.05(5.6–7.08)	− 0.078	0.938	1.003(0.845–1.189)	0.976
WBC,109/L	6.815(5.80–8.34)	6.875(5.92–8.5)	6.665(5.79–8.18)	− 1.043	0.297	1.115(0.992–1.253)	0.068
Neutrophil percentage(%)	63(56–70)	64(56–74)	63(57–68.75)	− 1.608	0.108	1.028(1.002–1.055)	0.037*
> = 72.5	44(19.6)	27(30.7)	17(12.5)	11.19	< 0.001***	3.098(1.568–6.121)	0.001**
Lymphocyte percentage(%)	27.5(21–33)	26(17–32.75)	29(22–33)	− 1.746	0.081	0.967(0.937–0.998)	0.037*
> = 19.5	176(78.6)	60(68.2)	116(85.3)	9.292	0.002**	0.369(0.192–0.71)	0.003**
Monocyte percentage(%)	7(6–8)	7(5–8)	7(6–8)	− 0.206	0.837	0.963(0.856–1.084)	0.531
Neutrophils, 109/L	4.36(3.35–5.51)	4.48(3.45–6.26)	4.27(3.33–5.22)	− 1.647	0.100	1.191(1.044–1.359)	0.009**
Neutrophils > = 5.01	78(34.8)	38(43.2)	40(29.4)	4.464	0.035*	1.824(1.042–3.194)	0.036*
Lymphocytes, 109/L	1.73(1.40–2.19)	1.67(1.31–2.17)	1.78(1.45–2.26)	− 1.048	0.295	0.818(0.545–1.229)	0.334
Monocytes, 109/L	0.46(0.35–0.61)	0.48(0.37–0.63)	0.45(0.35–0.6)	− 0.668	0.504	2.014(0.584–6.95)	0.268
Platelets, 109/L	208.5(186–247.5)	208(189.25–252)	209(183–243.25)	− 0.802	0.422	1.002(0.997–1.007)	0.429
NLR	2.31(1.73–3.41)	2.47(1.73–4.41)	2.18(1.73–2.95)	− 1.813	0.070	1.123(1.011–1.248)	0.031*
NLR > = 3.875	44(19.6)	27(30.7)	17(12.5)	11.19	< 0.001***	3.098(1.568–6.121)	0.001**
LMR	3.89(3–4.96)	3.66(2.6–4.94)	3.96(3.24–4.97)	− 1.519	0.129	1.004(1–1.008)	0.49
PLR	118.81(96.77–155.85)	135.58(97.04–165.02)	115.66(94.51–145.28)	− 1.577	0.115	0.943(0.799–1.113)	0.084
SII	493.65(351.69–751.19)	583.12(367.38–885.81)	471.05(348.3–698.24)	− 2.195	0.028*	1.001(1–1.001)*	0.017*
SII > = 767.3	52(23.2)	31(35.2)	21(15.4)	11.734	< 0.001***	2.978(1.573–5.64)	< 0.001***

Continuous variables are presented as median (25th–75th percentile), whereas categorical variables are presented as number (%) (*P < 0.05, **P < 0.01, ***P < 0.001). ^a^ The quantity of cardioembolism is too small, cardioembolism is included in others TOAST type for chi-square test.

*BMI* body mass index, *DWMH* deep white matter hyperintensities, *FBG* fasting blood glucose, *HDL* high-density lipoprotein, *LAA* large-artery atherosclerosis, *LDL* low-density lipoprotein, *LMR* lymphocyte-to-monocyte ratio, *NIHSS* National Institutes of Health Stroke Scale, *NLR* neutrophil-to-lymphocyte ratio, *PLR* platelet-to-lymphocyte ratio, *PSCI* post-stroke cognitive impairment, *PSCIN* post-stroke no cognitive impairment, *PVH* periventricular hyperintensity, *SII* Systemic Immune Inflammation Index, *TIA* transient ischemic attack, *TOAST* Trial of Org 10,172 in Acute Stroke Treatment.

## Peripheral immunity and PSCI

**Figure 2:**
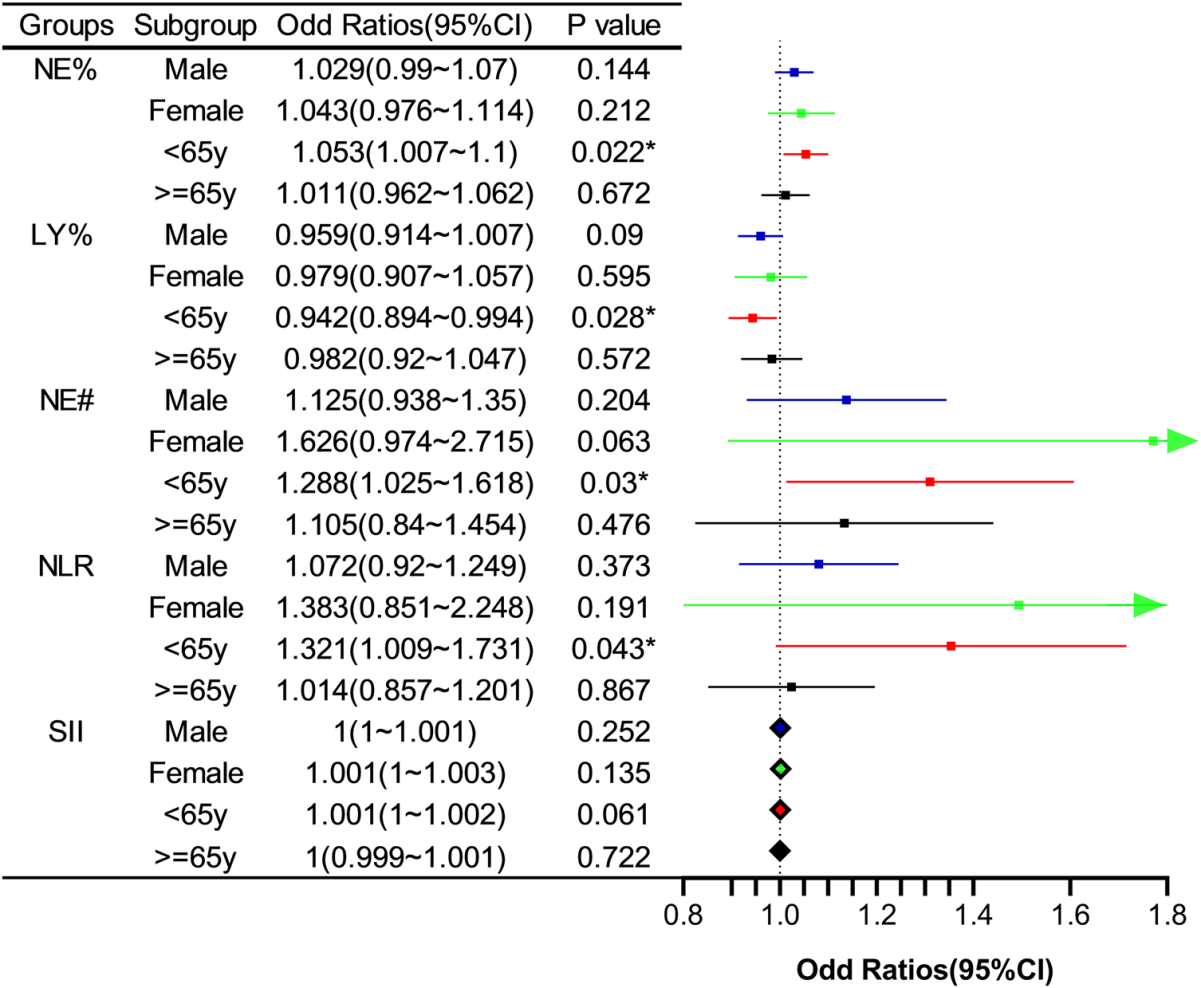
Associations between peripheral immunity, post-stroke cognitive impairment and dementia in age and sex subgroups (*P < 0.05). CI = confidence interval, LY% = lymphocyte percentage, NE% = neutrophil percentage, NE# = neutrophil value, NLR = neutrophil-to-lymphocyte ratio, SII = Systemic Immune Inflammation Index.

In the univariate logistic regression analysis, neutrophil percentage, neutrophil counts, NLR, and SII were found to significantly increase the risk of PSCI, whereas the lymphocyte ratio was associated with a lower risk of PSCI. However, monocytes, lymphocytes, platelets, PLR, and LMR had no significant effect on PSCI (Table 1). After adjusting for sex, age, height, systolic blood pressure, years of education, NIHSS score, DWMH score, Fazekas score, hypertension, and previous stroke, each 1% increase in the neutrophil ratio was related to a 3.6% increase in exposure to PSCI (odds ratio [OR], 1.036; 95% confidence interval [CI], 1.004–1.069; *P* = 0.025), and similar findings were observed for neutrophil values (per 109/L increment OR, 1.215; 95% CI, 1.03–1.432; *P* = 0.02). Each 1% increase in lymphocyte count lowered the risk of PSCI by 4.2% (OR, 0.958; 95% CI, 0.922–0.996; *P* = 0.029) (Table 2). Subgroup analyses were performed based on age and sex. In the 50–65-year-old subgroup, the risk of PSCI increased with an increasing neutrophil percentage (OR, 1.053; 95% CI, 1.007–1.1; *P* = 0.022), neutrophil values (OR, 1.288; 95% CI, 1.025–1.618; *P* = 0.03), and NLR (OR, 1.321; 95% CI, 1.009–1.731; *P* = 0.043), and a protective role was found for lymphocyte percentage (OR, 0.942; 95% CI, 0.894–0.994; *P* = 0.028). In the 65–80-year-old subgroup, none of the immunity indicators showed statistically significant differences for PSCI. In addition, none of the peripheral immune indicators showed any differences for PSCI in the subgroups stratified by sex (Fig. 2).

**Table 2:** Risk of post-stroke cognitive impairment according to peripheral immune markers.

	Unjusted	Model1	Model2	Model3
	OR(95%CI)	OR(95%CI)	OR(95%CI)	OR(95%CI)
NE%	1.028(1.002–1.055)*	1.03(1.003–1.059)*	1.037(1.006–1.068)*	1.036(1.004–1.069)*
LY%	0.967(0.937–0.998)*	0.963(0.931–0.995)*	0.957(0.922–0.992)*	0.958(0.922–0.996)*
MO%	0.963(0.856–1.084)	0.963(0.854–1.086)	0.957(0.841–1.089)	0.929(0.814–1.061)
NE#	1.191(1.044–1.359)**	1.221(1.062–1.404)**	1.244(1.066–1.452)**	1.215(1.03–1.432)*
LY#	0.818(0.545–1.229)	0.887(0.579–1.358)	0.834(0.526–1.321)	0.776(0.472–1.277)
MO#	2.014(0.584–6.95)	2.917(0.797–10.674)	2.784(0.67–11.565)	1.915(0.423–8.678)
PLT	1.002(0.997–1.007)	1.001(0.996–1.007)	1(0.994–1.006)	1(0.994–1.006)
NLR	1.123(1.011–1.248)*	1.121(1.004–1.252)*	1.129(0.995–1.282)	1.124(0.981–1.287)
LMR	0.943(0.799–1.113)	0.913(0.765–1.089)	0.931(0.77–1.125)	0.987(0.811–1.202)
PLR	1.004(1–1.008)	1.003(0.999–1.007)	1.003(0.998–1.007)	1.003(0.998–1.008)
SII	1.001(1–1.001)*	1.001(1–1.001)*	1.001(1–1.001)*	1.001(1–1.001)

Model 1 adjusts for age and sex; Model 2 adjusts for age, sex, years of education, and NIHSS score; and Model 3 adjusts for age, sex, years of education, height, systolic blood pressure, NIHSS score, DWMH score, Fazekas score, hypertension, and previous stroke (*P < 0.05, **P < 0.01, ***P < 0.001).

*CI confidence interval*, *DWMH* deep white matter hyperintensity, *LMR* lymphocyte-to-monocyte ratio, *LY*% lymphocyte percentage, *LY*# lymphocytes, *MO*% monocyte percentage, *MO*# monocytes, *NE*% neutrophil percentage, *NE*# neutrophils, *NIHSS* National Institutes of Health Stroke Scale, *NLR* neutrophil-to-lymphocyte ratio, *OR* odd ratios, *PLR* platelet-to-lymphocyte ratio, *PLT* platelets, *SII* Systemic Immune Inflammation Index.

## Prediction model for PSCI

**Figure 3:**
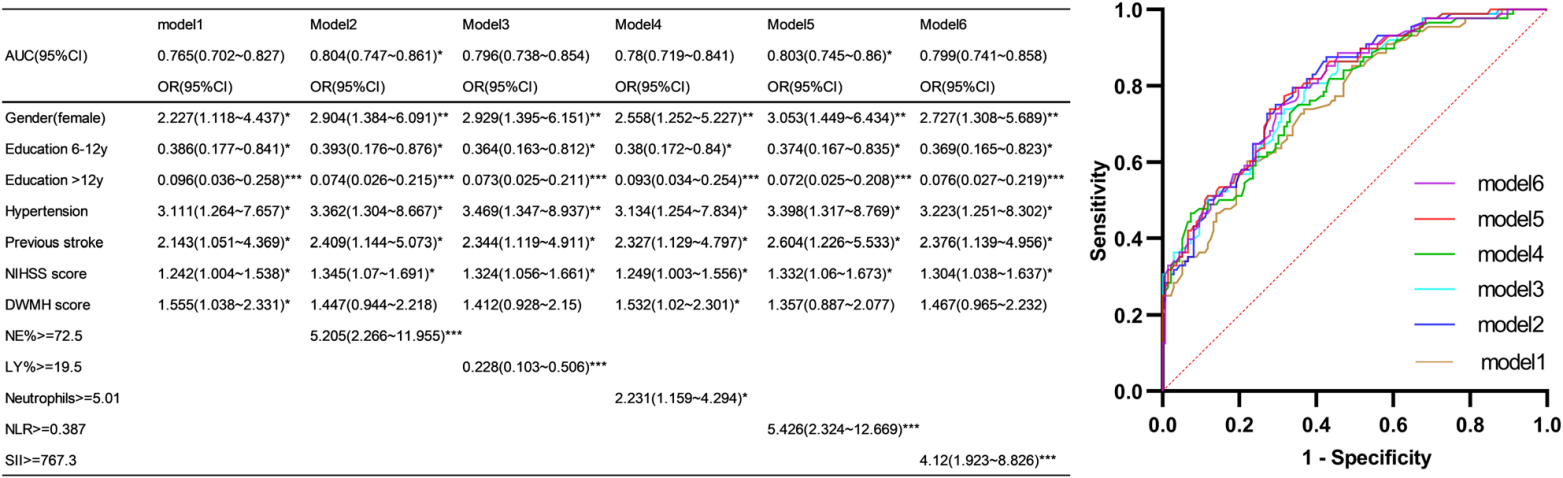
Prediction models and ROC curves. Model 1 is based on sex, education level, NIHSS score, hypertension, previous stroke, and DWMH score and serves as the reference model. Model 2 augments Model 1 with categorized neutrophil percentages. Model 3 extends Model 1 by adding categorized lymphocyte percentages. Model 4 expands Model 1 with the inclusion of categorized neutrophil values. Model 5 supplements Model 1 with categorized NLR. Model 6 supplements Model 1 with categorized SII. The cut-off values of NE%, LY%, neutrophils, NLR, and SII were derived from the optimal thresholding method based on the ROC curves (*P < 0.05, **P < 0.01, ***P < 0.001). AUC = area under the curve, CI = confidence interval, DWMH = deep white matter hyperintensity, LY% = lymphocyte percentage, NE% = neutrophil percentage, NIHSS = National Institutes of Health Stroke Scale, NLR = neutrophil-to-lymphocyte ratio, ROC = receiver operating characteristic, SII = Systemic Immune Inflammation Index, OR = odd ratio.

Due to the limited number of outcome events, after careful consideration, we developed a base prediction model for PSCI based on sex, education level, NIHSS score, hypertension, previous stroke, and DWMH score using meaningful indicators in stepwise backward multivariate binary logistic regression (Supplementary Table S1). The model showed that the area under the ROC curve was 0.765 when the neutrophil, lymphocyte percentage, neutrophil values, NLR, and SII levels in the model were 0.804, 0.764, 0.78, 0.803, and 0.799, respectively (Fig. 3). Compared to the base model, the model with a categorized neutrophil percentage and NLR significantly improved predictive efficacy (*P* = 0.042 and 0.049, respectively).

## Discussion

This prospective cohort study explored the association between peripheral immunity and PSCI in northeast China. The main results were as follows: 1) increased innate immunity markers (neutrophil percentage, neutrophil values, NLR, and SII) elevated the risk of PSCI, whereas elevated adaptive immunity markers (lymphocyte ratio) alleviated the risk of PSCI; 2) the influence of peripheral immunity on PSCI was most significant in the population aged 50–65 years; and 3) the neutrophil percentage and NLR significantly improved the predictive efficacy of PSCI.

A previous study based on 370,735 individuals from the UK Biobank suggested that increased neutrophils and NLR are positively associated with the incidence of vascular dementia incidence, while increased LMR is negatively related to it ^21^. However, only few studies conducted on peripheral immunity and PSCI, and the results are still controversial. Our previous cohort study found that a high neutrophil percentage and NLR are associated with PSCI at baseline ^25^, and a recent study suggested that SII is strongly related to cognitive impairment at the 3-month follow-up in patients with ischemic stroke ^31^. In line with the above results, one study of the systemic immune profiles in acute stroke survivors found that innate immune cell types increase in the acute phase (day 2), and adaptive immune cell types increase on day 5. Neutrophils and immunoglobulin M^+^ (IgM^+^) B cells remaine elevated until day 90, and the increase in peripheral innate immunity cells 2 days after stroke is closely associated with a decrease in MoCA scores at day 90 to 1-year follow-up time points, whereas the adptive immunity is not associated with these cognitive trajectories ^22^. However, peripheral immune-mediated delayed B-lymphocyte infiltration is assumed to be associated with the development of PSCI both in mice and human patients ^20^. Moreover, a study by Nguyen, V. A. et al. showed that elevated white blood cell and neutrophil counts within 1 week after stroke events are independently associated with better cognitive outcomes at 3 and 12 months ^32^. Our study systematically analyzed peripheral immunity and PSCI, providing insights for further research and potential treatments for PSCI.

The mechanisms underlying the effects of peripheral innate immunity on PSCI remain unclear. In a mouse stroke model with middle cerebral artery occlusion, neutrophils were continuously recruited from the blood to the brain 2 − 14 days after stroke ^33^. In the blood samples of stroke patients, neutrophils remained elevated 90 days after stroke ^22^. However, under homeostatic conditions and in early and late ischemic brain injury, neutrophils play different roles in the brain ^33^. In the acute stage, neutrophils interact with platelets and the vascular endothelium and release neutrophil extracellular traps, including matrix metalloproteinase 9, increasing blood–brain barrier permeability ^18^ and promoting further neutrophils infiltration. This is a vicious cycle ^18^ where neutrophils receptors activate the nuclear factor kappa B via pattern recognation, leading to the production of various cytokines ^10^. These interactions among different cell types, cytokines, and complement systems through different signaling pathways contribute to subsequent cognitive decline ^14,22^. Studies have shown that neutrophils have two major phenotypes, N1 and N2. The N1 phenotype can aggravate the injury within 3 days after stroke through the aforementioned mechanism, while the gradually increasing number of N2 phenotype can increase phagocytosis activity and promote the secretion of angiogenic factors, and play a role in anti-inflammatory response and tissue repair ^18,34^. This suggests the key role of neutrophils in the development of PSCI and may represent the underlying mechanism whereby increased neutrophils within 24h of acute ischemic events increase the incidence of PSCI observed in our study.

Adaptive immunity is also a multifaceted process. In an intraluminal filament middle cerebral artery occlusion model, B-cell-deficient mice showed more severe functional deficits within the first 48h after stroke than wild-type mice, suggesting that regulatory B lymphocytes are beneficial during acute stroke events ^35^. Consistent with the above study, our study suggests that an increase in the lymphocyte ratio in the acute stroke phase is protective against cognitive impairment 6–12 months later. However, Doyle et al. demonstrated the crucial role of B lymphocytes in developing cognitive impairment 7 weeks later in the mouse model of post-stroke dementia (PSD) ^19^. Their immunostaining results of postmortem brains of patients with stroke suggested that B-lymphocyte infiltration contributes to PSCI and PSD ^19^. Weitbrecht et al. provided further evidence that lymphocyte infiltration is a long-term ongoing process after stroke, and that the delayed response of B cells promoted by CD4 + T cells and CD4 depletion improved cognitive function in experimental ischemic stroke mice ^36^. The conflicts between these results may be attributable to different sampling time points and sampling sites of observation, as the B cell phenotype may be beneficial or detrimental depending on the subset, timing, and microenvironment ^37^. Further research is required to investigate the complex roles of adaptive immunity in PSCI. Studies in mice conducted by Doyle et al. suggested that B lymphocytes interacted with thymus-independent type 2 antigens to form natural IgA antibodies, which were similar to those secreted by the intestinal mucosa. These IgA + plasma cells were thought to infiltrate the damaged brain tissue, leading to PSCI ^20^. Further animal experiments suggested that anti-inflammatory drug fingolimod is a promising therapy for PSCI ^16^. Microbiota dysbiosis after stroke can affect neuroinflammation and peripheral immunity by altering the lymphocyte population ^38^. Both animal and human experiments have confirmed that stroke-induced intestinal flora disturbance is associated with PSCI, and animal experiments supplemented with butyrate can improve post-stroke cognitive function ^39^. Notably, both vegan and ketogenic diets can significantly affect the microbiota in the human body, up regulating the innate and adaptive immune functions, respectively ^40^. The development of drugs that regulate the gut microbiota could be a very promising treatment for PSCI. However, owing to the complex immunomodulatory response described above, disease-modifying treatments for patients with PSCI are still far from animal experiments to clinical translation ^22,23^. Acupuncture has also been reported to improve cognitive impairment in vascular dementia by modulating peripheral immunity ^41^. Further research is warranted to fully clarify its impact on PSCI.

Our prospective cohort study, which included patients with 6–12 months of follow-up data, provided further evidence regarding the relationship between immunity and PSCI, as well as a novel perspective for exploring the etiology and further treatment of PSCI. However, this study has several limitations. First, it was conducted at a single center including patients with TIA or minor stroke aged 50–80 years, with a follow-up of 6–12 months. Because of the global outbreak of COVID-19 that occurred during the study period, only 224 patients were followed up on time. We found that patients who were followed up had a better education, better cognitive function, higher mononuclear ratio, lower homocysteine, lower Fazekas and PVH scores, lower prevalence of diabetes, lower systolic blood pressure, and differences in stroke type compared with patients who were not followed up with (Supplementary Table S3). Larger samples from multicenter studies are required. Second, we did not assess cognitive function and peripheral immunoinflammatory indicators before the stroke, although we collected detailed medical histories to exclude patients with a history of cognitive impairment and drug/disease-related changes in cognition and peripheral immunoinflammatory indicators. Third, different studies define minor stroke according to the NIHSS scores, as follows: ≤ 3 ^7^ , ≤ 5 points ^42^ , and ≤ 6 ^43^ points. In order to make our study more applicable, we chose NIHSS scores ≤ 6 as our criterion for diagnosing minor stroke. Since studies concerning the treatment and prognosis of TIA and minor stroke have almost always been conducted together ^42^, and because of the limited sample size in this study, we did not categorize our patients into stroke and TIA groups or analyze them separately based on cognitive level and divided them into PSCI and PSD groups. Finally, there is no global consensus on when and how to diagnose PSCI, but it is generally accepted that it occurs after stroke events and persists for ≥ 6 months afterward ^3^. Here, we used MoCA scores within 6–12 months of follow-up as a reference for diagnosis and did not exclude patients with recurrent stroke. Further research is needed to investigate the roles of different peripheral immune indicators in PSCI at different time points after stroke.

In conclusion, we conducted a comprehensive analysis of peripheral immunity in patients with PSCI, and our results suggest that routine peripheral blood testing should be performed as soon as possible after acute brain ischemia, especially within the first 24h, which has great predictive value for PSCI. Elevated in the neutrophil ratios, neutrophil counts, NLR, and SII values exacerbates post-stroke cognitive impairment. In contrast, an increased lymphocyte count plays a protective role against PSCI. An increased neutrophil count in the acute phase of TIA and ischemic stroke will likely trigger PSCI. The neutrophil percentage and NLR, combined with demographic and clinical information, significantly improved the predictive value, which can assist in early detection and treatment of PSCI. Large-scale multicenter clinical studies are warranted to further investigate peripheral immunity mechanisms in PSCI and their associated interventions over different follow-up periods after acute brain ischemic events.

### Supplementary Information

Supplementary Figure 1.

Supplementary Legends.

Supplementary Tables.

## Data Availability

The relevant data are under the Patient Privacy Protection Treaty, and relevant statistics, if justified, can be obtained by contacting the corresponding author.

## Abbreviations

AD: Alzheimer’s disease
CI: Confidence interval
COVID-19: Coronavirus disease 2019
DWMH: Deep white matter hyperintensity
LMR: Lymphocyte-to-monocyte ratio
MMSE: Mini-Mental State Examination
MoCA: Montreal Cognitive Assessment
NIHSS: National Institutes of Health Stroke Scale
NLR: Neutrophil-to-lymphocyte ratio
OR: Odds ratio
PLR: Platelet-to-lymphocyte ratio
PSCI: Post-stroke cognitive impairment
PVH: Periventricular hyperintensity
ROC: Receiver operating characteristic
SII: Systemic immune inflammation index
TOAST: Trial of Org 10172 in Acute Stroke Treatment
