# Supplementary figures and images for: Peripheral immunity is associated with cognitive impairment after acute minor ischemic stroke and transient ischemic attack
Source: Sci Rep. 2024 Jul 13;14:16201. doi: 10.1038/s41598-024-67172-w (PMC11246473; doi:10.1038/s41598-024-67172-w)

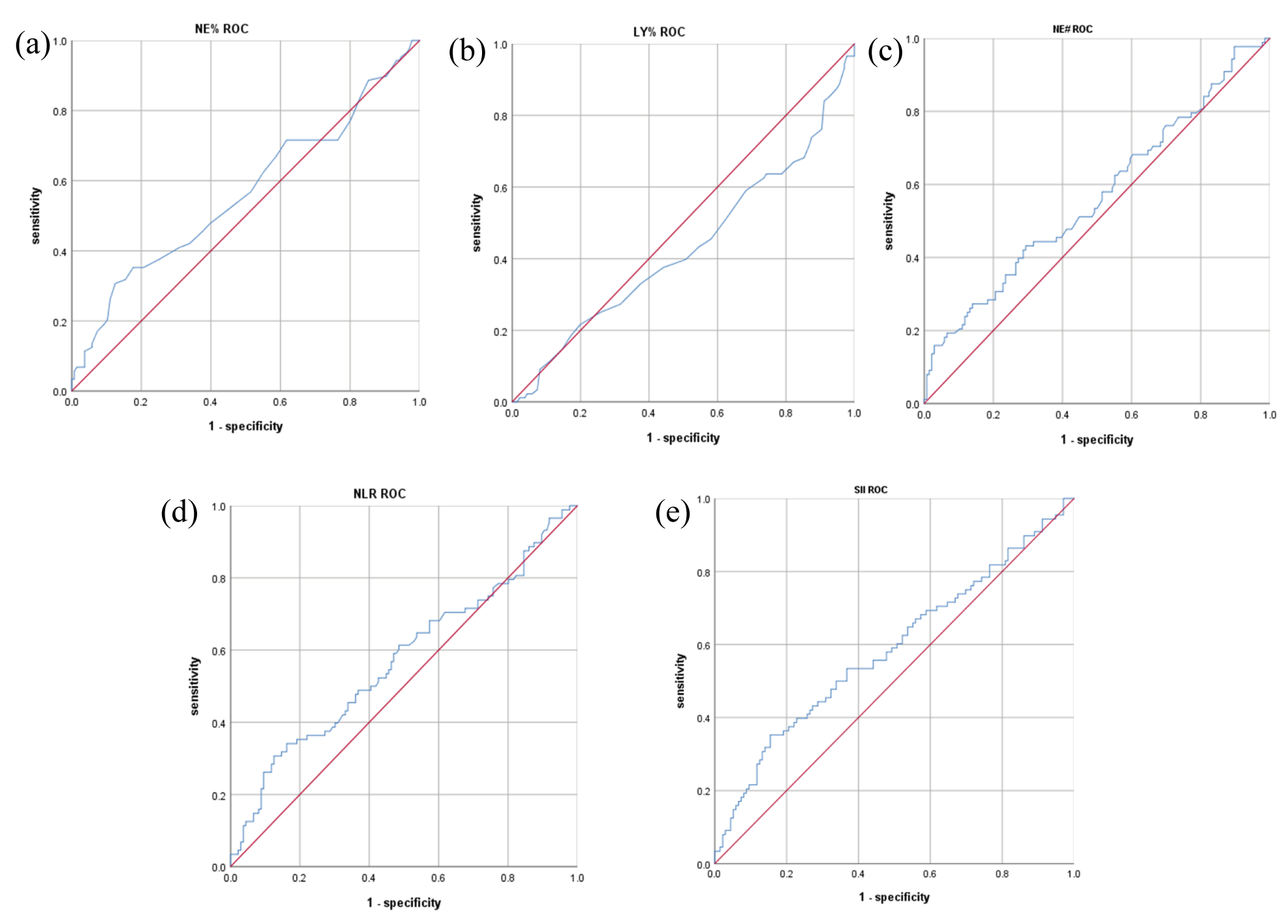

Supplement: Supplementary file 1 — Supplementary Figure 1. [file 41598_2024_67172_MOESM1_ESM.tif]
