# Supplementary material for: Peripheral immunity is associated with cognitive impairment after acute minor ischemic stroke and transient ischemic attack
Source: Sci Rep. 2024 Jul 13;14:16201. doi: 10.1038/s41598-024-67172-w (PMC11246473; doi:10.1038/s41598-024-67172-w)
Supplement: Supplementary file 2 — Supplementary Legends. [file 41598_2024_67172_MOESM2_ESM.docx]

**Supplementary Informations**

**Peripheral Immunity is Associated with Cognitive Impairment after Acute Minor Ischemic Stroke and Transient Ischemic Attack**

PanPan Zhao; GuiMei Zhang; YongChun Wang; ChunXiao Wei; ZiCheng Wang; WeiJie Zhai; YanXin Shen; Lin Shi; Li Sun *

Department of Neurology and Neuroscience Center, The First Hospital of Jilin University, Jilin University, Changchun, China

* Corresponding author: Li Sun (ORCID 0000-0003-1169-4653)

Department of Neurology and Neuroscience Center, The First Hospital of Jilin University, Jilin University, Xinmin Street 71#, Changchun 130021, China

Telephone: +86-13504313689

Fax: +86-431-88782378

E-mail address: [sunli99@jlu.edu.cn](mailto:sunli99@jlu.edu.cn)

**Supplementary materials**

**Supplementary Fig. S1** The cut-off values of neutrophil percentage, lymphocyte percentage, neutrophils, NLR, and SII

(a) The area under the receiver operating characteristic curves (AUC) of NE% was 0.564 , and the cutoff value was 72.5%;

(b) The AUC of LY% was 0.431, and the cutoff value was 19.5%;

(c) The AUC of NE# was 0.565, and the cutoff value was 5.01×109/L;

(d) The AUC of NLR was 0.572, and the cutoff value was 3.875;

(e) The AUC of SII was 0.587, and the cutoff value was 767.3.

Abbreviations: LY% = lymphocyte percentage, NE% = neutrophil percentage, NE# = neutrophil counts, NLR = neutrophil-to-lymphocyte ratio, ROC= receiver operating characteristic, SII = Systemic Immune Inflammation Index.

**Supplementary Table S1**: Univariate and multivariate regression analysis of statistically significant variables

^a^The base prediction model for PSCID is based on sex, education level, NIHSS score, hypertension, previous stroke, and DWMH score using meaningful indicators in stepwise backward multivariate binary logistic regression due to the limited number of outcome events, ^b^y=year.

Abbreviations: CI = confidence interval, DWMH = deep white matter hyperintensities, LAA = large

artery atherosclerosis, NIHSS= National Institutes of Health Stroke Scale, OR= odd ratio, SBP=

systolic pressure, TOAST = Trial of Org 10172 in Acute Stroke Treatment.

**Supplementary Table S2**: The missing data

Abbreviations: DWMH = deep white matter hyperintensities, FBG = fasting blood glucose, HDL = high-density lipoprotein, LDL = low-density lipoprotein.

**Supplementary Table S3**: Bias analysis between followed-up and non-followed-up patients

Continuous variables are presented as median (25th–75th percentile), whereas categorical variables are presented as numbers (%).

Abbreviations: BMI = body mass index, DWMH = deep white matter hyperintensities, FBG = fasting blood glucose, HDL = high-density lipoprotein, LAA = large-artery atherosclerosis, LDL = low-density lipoprotein, LMR = lymphocyte-to-monocyte ratio, MMSE= Mini-Mental State Examination, MOCA= Montreal Cognitive Assessment, NIHSS= National Institutes of Health Stroke Scale, NLR = neutrophil-to-lymphocyte ratio, PLR = platelet-to-lymphocyte ratio, PVH = periventricular hyperintensity, SII = Systemic Immune Inflammation Index, TIA = transient ischemic attack, TOAST = Trial of Org 10172 in Acute Stroke Treatment.
